# Relationship Between Internet Use and Cognitive Function Among Middle-Aged and Older Chinese Adults: 5-Year Longitudinal Study

**DOI:** 10.2196/57301

**Published:** 2024-12-02

**Authors:** Bowen Chen, Chun Yang, Shanshan Ren, Penggao Li, Jin Zhao

**Affiliations:** 1 Department of Hospital Epidemiology and Infection Control Sir Run Run Shaw Hospital Zhejiang University School of Medicine Hangzhou China; 2 School of Public Health Capital Medical University Beijing China; 3 Department of Clinical Nutrition Beijing Hospital Chinese Academy of Medical Sciences Beijing China

**Keywords:** aging, cognitive function, internet use, longitudinal study, fixed effects model

## Abstract

**Background:**

Cognitive decline poses one of the greatest global challenges for health and social care, particularly in China, where the burden on the older adult population is most pronounced. Despite the rapid expansion of internet access, there is still limited understanding of the long-term cognitive impacts of internet use among middle-aged and older adults.

**Objective:**

This study aims to explore the association between internet use and age-related cognitive decline among middle-aged and older Chinese adults. To gain a more comprehensive understanding of the effects of internet use, we also focused on assessing the impact of both the frequency of internet use and the types of internet devices on cognition. Moreover, we assessed the mediating role of internet use on cognitive function for characteristics significantly linked to cognition in stratified analysis.

**Methods:**

We analyzed data based on 12,770 dementia-free participants aged ≥45 years from the China Health and Retirement Longitudinal Study. We used a fixed effects model to assess the relationship between internet use and cognitive decline and further validated it using multiple linear regression analysis, generalized estimating equations, propensity score matching, inverse probability of treatment weighting, and overlap weighting. We further examined the varying effects of internet device type and frequency on cognitive function using fixed effects models and Spearman rank correlations. The Karlson-Holm-Breen method was used to estimate the mediating role of internet use in the urban-rural cognitive gap.

**Results:**

Participants using the internet (n=1005) were younger, more likely to be male, more educated, married, retired and living in an urban area and had higher cognitive assessment scores than nonusers (n=11,765). After adjusting for demographic and health-related risk factors, there was a positive correlation between internet use and cognitive function (β=0.551, 95% CI 0.391-0.710). Over the follow-up period, persistent internet users had a markedly lower 5-year incidence of neurodegenerative diseases, at 2.2% (15/671), compared with nonusers, at 5.3% (379/7099; *P*<.001). The negative impact of aging (>50 years) on cognitive function was consistently less pronounced among internet users than among nonusers. Furthermore, increased frequency of internet use was associated with greater cognitive benefits for middle-aged and older adults (r_s_=0.378, *P*<.001). Among digital devices used for internet access, cell phones (β=0.398, 95% CI 0.283-0.495) seemed to have a higher level of cognitive protection than computers (β=0.147, 95% CI 0.091-0.204). The urban-rural disparity in cognitive function was partially attributed to the disparity in internet use (34.2% of the total effect, *P*<.001).

**Conclusions:**

This study revealed that the use of internet by individuals aged 45 years and older is associated with a reduced risk of cognitive decline. Internet use has the potential to be a viable, cost-effective, nonpharmacological intervention for cognitive decline among middle-aged and older adults.

## Introduction

With the rapid increase in the aging population and the extension of life expectancy, age-related cognitive impairment has emerged as an influential global public health concern [1]. Cognitive decline is an irreversible cerebral pathological and physiological progression, serving as both a precursor to dementia and a potential catalyst for physical disability and mortality [2]. A nationwide survey conducted in 2019 revealed that China has become the country with the highest number of individuals with dementia, as the population aged 60 years and older has soared to a staggering 253 million people, among which 6.0% have dementia [3,4]. Cognitive impairment typically emerges in individuals aged 45 years to 60 years and not only imposes a significant burden on the patients’ families but also presents formidable challenges to the health care system and society [5]. Hence, the discovery of nonpharmacological strategies to intervene for middle-aged and older adults is crucial to reducing the incidence of cognitive decline, slowing the progression of cognitive impairment, and improving health and well-being.

Regarding the prevention of dementia, establishing a healthy lifestyle and implementing nonpharmacological interventions are critically important. Based on randomized clinical trials and meta-analyses, consistent research-based evidence indicates that social and cognitively stimulating activities can delay cognitive decline, particularly in middle-aged and older adults who are most susceptible to cognitive impairment [6-8].

With the rapid development of the internet and the widespread use of smartphones, digital literacy is increasing in China, particularly among middle-aged and older adults. According to the 52nd Statistical Report on Internet Development in China, released by the China Internet Network Information Center [9], the number of internet users aged 60 years and older reached a staggering 140 million as of June 2023. It signifies a profound penetration of internet use within the older adult demographic, bringing about substantial transformations in daily life. Preliminary analysis of the English Longitudinal Study of Aging indicated that individuals who frequently use the internet or email exhibit a 3.1% improvement in delayed recall compared with those who do not use the internet or email [10]. An Australian study revealed that men aged 70 years and older who use the internet had a 50% relative risk reduction for dementia, even after controlling for age, education, social relationships, and physical health status [11]. The study by Klimova [12] suggested that the use of the internet, specifically online cognitive training programs, may exert a beneficial effect on enhancing the cognitive function of healthy older adults.

The mechanisms through which internet use enhances cognitive abilities are highly complex, and current research has not fully elucidated them yet. A cross-sectional study showed that internet searches can enhance the activation of neural circuits in the frontal pole, anterior temporal region, anterior and posterior cingulate, and hippocampus areas among people older than 55 years [13]. Numerous previous studies have indicated that the internet can provide people with more social contacts, social support, and positive attitudes toward life, enhancing social connections while reducing loneliness and social isolation, which can have a positive impact on cognitive function [14-16]. The study by Firth et al [17] suggested that the use of the internet may influence cognitive function through 3 key factors: attentional capacity, memory processes, and social cognition.

However, not all research findings consistently demonstrate the cognitive benefits of internet use. A randomized controlled trial conducted in the Netherlands with a small sample of individuals aged 64 years to 75 years revealed that 12 months of computer and internet use did not have a significant impact on the cognitive function of older adults [18]. Overall, despite the common perception that internet use can improve cognitive function in older adults, the current literature provides varying evidence, necessitating studies with larger sample sizes to elucidate the correlation.

The China Health and Retirement Longitudinal Study (CHARLS) is the first nationally representative survey of middle-aged and older adults in China, with a sample collected from 28 provinces and 150 regions across the country [19]. This project aims to construct a high-quality public microdatabase collecting multidimensional information encompassing socioeconomic status and health conditions to meet the needs of gerontological research. Therefore, the CHARLS questionnaire includes several aspects such as demographic background, family structure, health status, health insurance, work, retirement, pensions, income, expenditures, assets, and house property. To ensure randomization and the representativeness of the sample, CHARLS adopts multistage stratified probability proportional to size sampling. Meanwhile, CHARLS conducts face-to-face interviews facilitated by well-trained surveyors and implements effective quality control through methods such as data verification, audio playback, and the use of a computer-assisted personal interview system.

Our primary aim was to assess the independent association between internet use and cognitive function in middle-aged and older adults by collecting survey data from the CHARLS study. To gain deeper insights into the cognitive effects of internet use, we also evaluated the age-related impacts on cognitive function in internet users and nonusers as one of our secondary objectives. Additionally, to explore the most effective means for enhancing cognitive function, we analyzed the correlations between cognitive function and both internet device use and frequency. We further intended to evaluate the mediating role of internet use on cognitive function for participant characteristics for which a significant association between internet use and cognitive function was observed in stratified analysis.

## Methods

### Participants

Participants aged 45 years and older were enrolled in the CHARLS study and included in subsequent secondary analyses, with their socioeconomic and health status being systematically recorded. The CHARLS survey was initiated in 2011 (Wave 1), followed by 4 consecutive waves of regular follow-up questionnaires conducted in 2013 (Wave 2), 2015 (Wave 3), 2018 (Wave 4), and 2020 (Wave 5). More comprehensive information on the study design and sampling strategies used in CHARLS has been previously described [19]. Given the insufficient number of internet users in Waves 1 and 2, we conducted data collection for the subsequent waves, including Wave 3, Wave 4, and Wave 5.

### Measurements

#### Internet Use

The primary exposure and mediating variable was internet use, which was assessed based on a binary response (yes/no) to the question regarding internet access. With regard to the frequency of internet use, participants’ questionnaire responses were classified into 4 categories: never, infrequently, weekly, and daily. Additionally, the devices used for internet access were categorized into 2 groups: computers (including desktop computers, laptop computers, and tablet computers) and cell phones. The specific wording of the questionnaire survey on internet use is detailed in Multimedia Appendix 1.

#### Cognitive Function

We used 2 established composite measures, mental intactness and episodic memory, to evaluate global cognitive function. These measures are similar to the assessment indicators used by the Health and Retirement Study in the United States [20] and have been validated among the Chinese population [21]. Mental intactness is determined based on 3 indicators of cognitive status, which comprise time orientation (naming month, day, year, week, and season), numerical ability (subtracting 7 consecutively from 100, 5 times), and visual and spatial abilities (the task of redrawing 2 overlapping pentagons). The mental intactness score for the 3 elements ranges from 0 to 11. Episodic memory can be evaluated through the recall of commonly used phrases, including immediate word memory (score range 0-10) and delayed word memory (score range 0-10, with a total score of 20). Cognitive function is constructed from these 2 components (Multimedia Appendix 1), with higher scores suggesting better cognitive function. Furthermore, to gain a more comprehensive understanding of cognitive benefits, we analyzed the incidence of neurodegenerative diseases, such as Alzheimer disease and Parkinson disease, among both internet users and nonusers during the follow-up period.

#### Covariates

This study accounted for multiple potential confounding factors that are known risk factors for cognitive function [22], in order to provide more compelling evidence. In our study, we took into account the following covariates: baseline or prior cognitive score (continuous variable), gender (male or female), age (continuous variable), marital status (yes [married or partnered] or no [unmarried, separated, divorced, widowed]), residency (urban or rural), retirement status (yes or no), education level (illiterate, low education level [primary or junior high school], mid education level [senior high school], or high education level [college or above]), smoking status (current smoker, former smoker, never smoked), alcohol consumption (never drank, drink less than once a month, drink more than once a month), diabetes (yes or no), hypertension (yes or no), and per capita household expenditure (quartiles).

### Statistical Analysis

To comprehensively assess the relationship between internet use and cognitive function, we used both longitudinal and cross-sectional analytical approaches, thereby aiming to complement the limitations inherent in each research method. We used fixed effects analysis to analyze longitudinal data with time-varying variables and performed multiple linear regression analyses on cross-sectional data from each wave. Furthermore, we used the restricted cubic spline regression method to investigate the differences in cognitive performance between internet users and nonusers across various ages. We used the Spearman rank correlation test to analyze the correlation between internet use frequency and cognitive function. We regarded baseline characteristics as covariates and conducted stratified and interaction analyses to assess the potential differences in the impact of internet use among different subgroups. After stratifying by baseline characteristics, we constructed a fixed effects model and incorporated the cross-product terms (internet use × baseline characteristics) within the same model to evaluate the interaction between internet use and baseline characteristics.

The mediation analysis was performed using the Karlson-Holm-Breen (KHB) method [23], which dissects the influence of urban-rural areas on cognitive functionality into its distinct direct and indirect constituents. In particular, it measures the extent to which the intermediate variable (internet use) mediates the association between the independent variable (urban-rural status) and the dependent variable (cognitive function).

In sensitivity analysis, we conducted propensity score matching (PSM) [24] with a 1:1 ratio to adjust for covariates and analyze the impact of internet use on cognitive function. Second, we also used inverse probability of treatment weighting (IPTW) [25] and overlap weighting (OW) [26] methods to weight the balance based on covariates, aiming to validate our main results. To obtain robust results and further enhance the sensitivity analysis, we additionally selected individuals who had been continuous internet users and continuous nonusers during a 5-year follow-up period to investigate their impact on cognitive function using generalized estimating equations (GEE) and multiple linear regression analysis. Continuous variables are shown as means and SDs, while categorical variables are represented as numbers (proportions). All analyses were performed using R (version 4.0.2) and Stata (version 14.1).

### Ethical Considerations

The Biomedical Ethics Review Committee of Peking University approved the ethical application (IRB00001052-11014 and IRB00001052-11015) for collecting data from human participants in all waves, as well as for secondary analysis of CHARLS data. Written informed consent was obtained from all participants prior to the questionnaire survey, and the original informed consent allows secondary analysis without additional consent. All respondents who completed the questionnaire received cash as compensation according to the unified standard amount set by the CHARLS in advance. In order to protect the privacy of the respondents, all the data in this study are anonymous.

## Results

### Descriptive Statistics

**Figure 1:**
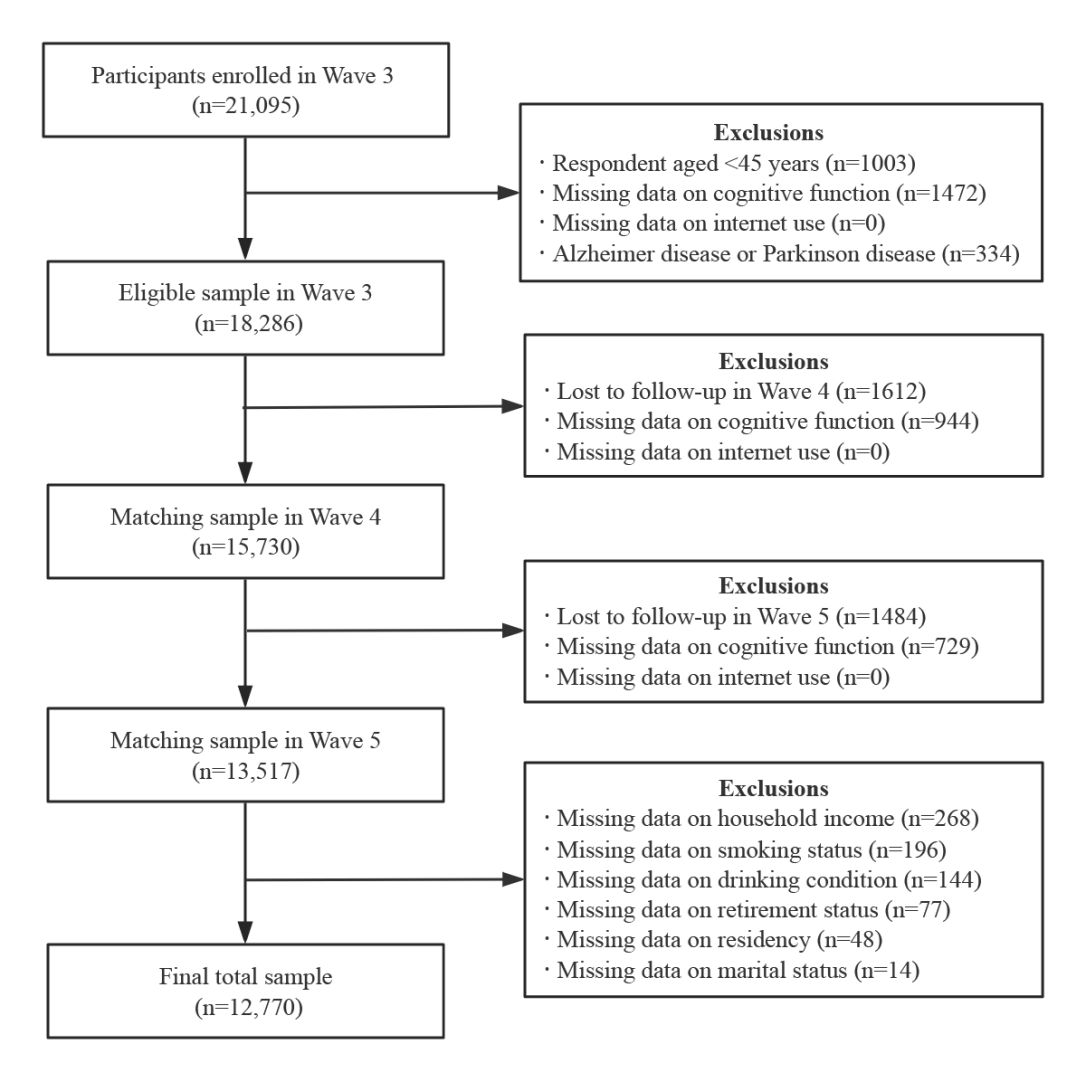
Flowchart of participant selection.

A total of 12,770 Chinese adults aged 45 years and older were ultimately included in this study, with the process of selection and exclusion detailed in Figure 1. In addition, Table 1 presents the baseline (ie, Wave 3) demographic characteristics of the study participants. At baseline, internet users accounted for 7.9% (1005/12,770) of the participants, and nonusers accounted for 92.1% (11,765/12,770) of the participants. Participants engaging in internet use had a significantly lower mean age of 53.04 (SD 7.33) years in contrast to the 59.32 (SD 9.16) years among nonusers (*P*<.001). More men were internet users (583/1005, 58%) than nonusers (5507/11,765, 46.8%). In terms of educational attainment, the internet-using population had a markedly lower illiteracy rate of 0.6% (6/1005) than the nonuser population (2269/11,765, 19.3%). This trend was consistent across educational strata, with 11.2% (113/1005), 71.9% (722/1005), and 16.3% (164/1005) of internet users classified as having low, mid, and high levels of education, respectively, as opposed to 44.1% (5189/11,765), 35.3% (4159/11,765), and 1.3% (148/11,765), respectively, of the nonuser population. Marital status among internet users was predominantly characterized by marriage, with 93.9% (944/1005) of this group being married, which was a higher proportion than the 90.3% (10,623/11,765) of nonusers who were married (*P*<.001). The proportion of retired individuals was also higher within the internet-using group, at 21% (211/1005) compared with 9.1% (1072/11,765) among nonusers (*P*<.001). Residence in rural areas was less common among internet users, with 46.9% (471/1005) living in such regions, a significantly lower proportion than the 83.5% (9825/11,765) of nonusers (*P*<.001). Notably, in terms of cognitive function, the cognitive assessment scores of internet users across different age groups (45-54 years, 55-64 years, 65-74 years, and 75 years) were significantly higher than those of nonusers (*P*<.001).

**Table 1:** Baseline characteristics of the study population.

Characteristic		Nonusers (n=11,765)	Internet users (n=1005)	*P* value	
**Gender** **, n (%)**					<.001
	Male	5507 (46.8)	583 (58)		
	Female	6258 (53.2)	422 (42)		
Age (years), mean (SD)		59.32 (9.16)	53.04 (7.33)	<.001	
**Age group (years), n (%)**					<.001
	45-54	4384 (37.3)	692 (68.8)		
	55-64	3917 (33.3)	219 (21.8)		
	65-74	2754 (23.4)	84 (8.4)		
	≥75	710 (6)	10 (1)		
**Household income per capita^a^, n (%)**					<.001
	Quartile 1	2779 (23.6)	137 (13.6)		
	Quartile 2	2877 (24.4)	146 (14.5)		
	Quartile 3	3091 (26.3)	250 (24.9)		
	Quartile 4	3018 (25.7)	472 (47)		
**Marital** **status, n (%)**					<.001
	Yes	10,623 (90.3)	944 (93.9)		
	No	1142 (9.7)	61 (6.1)		
**Residency, n (%)**					<.001
	Rural	9825 (83.5)	471 (46.9)		
	Urban	1940 (16.5)	534 (53.1)		
**Educational attainment, n (%)**					.001
	Illiterate	2269 (19.3)	6 (0.6)		
	Low education	5189 (44.1)	113 (11.2)		
	Mid education	4159 (35.3)	722 (71.9)		
	High education	148 (1.3)	164 (16.3)		
**Retirement status, n (%)**					<.001
	No	10,693 (90.9)	794 (79)		
	Yes	1072 (9.1)	211 (21)		
**Smoking status, n (%)**					<.001
	Never smoked	4533 (38.5)	294 (29.3)		
	Quit	2234 (19)	195 (19.4)		
	Still smoke	4998 (42.5)	516 (51.3)		
**Drinking status, n (%)**					<.001
	Never drank	7579 (64.4)	432 (43)		
	Drink less than once a month	996 (8.5)	195 (19.4)		
	Drink more than once a month	3190 (27.1)	378 (37.6)		
**Hypertension, n (%)**					.045
	No	8353 (71)	744 (74)		
	Yes	3412 (29)	261 (26)		
**Dyslipidemia, n (%)**					.12
	No	8526 (72.5)	752 (74.8)		
	Yes	3239 (27.5)	253 (25.2)		
**Diabetes, n (%)**					.004
	No	8387 (71.3)	760 (75.6)		
	Yes	3378 (28.7)	245 (24.4)		
**Cognitive assessment score by age (years), mean (SD)**					
	45-54	16.00 (4.85)	19.99 (3.61)	<.001	
	55-64	14.50 (4.98)	19.69 (3.30)	<.001	
	65-74	13.49 (5.20)	19.24 (3.67)	<.001	
	≥75	11.46 (5.25)	18.00 (4.71)	<.001	

^a^Divided into 4 quartiles based on the 25th percentile (¥1643.60 [US $226.89]), 50th percentile (¥4497.50 [US $620.86]), and 75th percentile (¥10,959.80 [US $1512.95]) of the overall data.

### Association Between Internet Use and Cognitive Function

**Figure 2:**
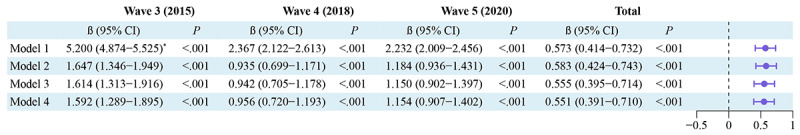
Associations between cognitive function and internet use. *Crude model.

This study demonstrated a positive association between internet use and cognitive function among middle-aged and older adults (Figure 2) based on the multiple linear regression analysis to evaluate cross-sectional data from Wave 3, Wave 4, and Wave 5 and the fixed effects model to analyze the longitudinal data across the entire population. Model 1 was adjusted for baseline or prior cognitive score; Model 2 was additionally adjusted for age, gender, marital status, educational attainment, residency, and retirement status; Model 3 was additionally adjusted for smoking, drinking, hypertension, and diabetes; and Model 4 was additionally adjusted for household income per capita. There were significant differences between internet users and nonusers in both mental intactness score and episodic memory score (*P*<.001; Multimedia Appendix 2). In the fixed effects model adjusting only for prior cognitive scores, internet use was significantly associated with cognitive function (model 1: β=0.573, 95% CI 0.414 to 0.732). The relationship remained statistically significant for model 2 (β=0.583, 95% CI 0.424 to 0.743) and model 3 (β=0.555, 95% CI 0.395 to 0.714). In the fully adjusted model (model 4), which additionally controlled for household income per capita, internet use remained positively associated with cognitive function (β=0.551, 95% CI 0.391 to 0.710). To further confirm the cognitive benefits of internet use, we conducted a multiple linear regression analysis on the waves of each cross-sectional data. Even after adjusting for all covariates, the results remained consistent with our expectations (Wave 3: β=1.592, 95% CI 1.289 to 1.895; Wave 4: β=0.956, 95% CI 0.720 to 1.193; Wave 5: β=1.154, 95% CI 0.907 to 1.402).

To further investigate the association between internet use and specific clinical manifestations of cognitive function, we analyzed the incidence of neurodegenerative diseases (Alzheimer disease or Parkinson disease) among internet users and nonusers (Multimedia Appendix 3). Notably, the 5-year incidence of neurodegenerative diseases was significantly lower in persistent internet users (15/671, 2.2%) than in nonusers (379/7099, 5.3%; *P*<.001), as detailed in Multimedia Appendix 3. This implies a potential association between internet use and the prevention of neurodegenerative diseases.

### Impact of Internet Use on Cognitive Function Across Age

**Figure 3:**
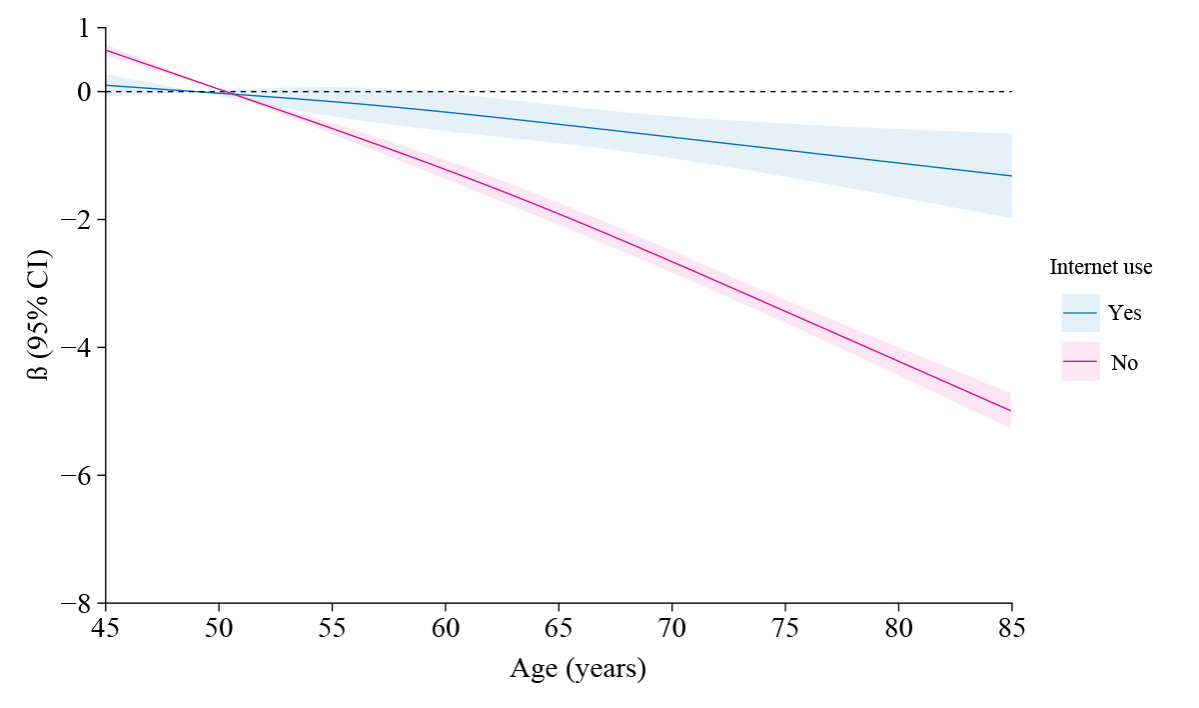
The influence of internet use on cognitive function across different ages using restricted cubic splines with 3 knots.

In order to further investigate the effects of internet use on cognitive function across different ages, we used restricted cubic splines to fit the curves that influence cognitive function (Figure 3). Our results indicated that age becomes a risk factor for cognitive function in internet nonusers after the age of 50.02 years, whereas for internet users, it occurs after the age of 49.02 years. Interestingly, we observed that the impact of age on cognitive decline was consistently minor among internet users compared with among nonusers after the age of 50 years. In other words, internet use has the potential to slow age-related cognitive decline. The results also showed that the older the age, the larger the gap in the effect of age on cognitive function between internet users and nonusers. This implies that the protective effect of internet use on cognitive decline is increasingly evident with age.

### Impact of Internet Device and Frequency on Cognitive Function

**Figure 4:**
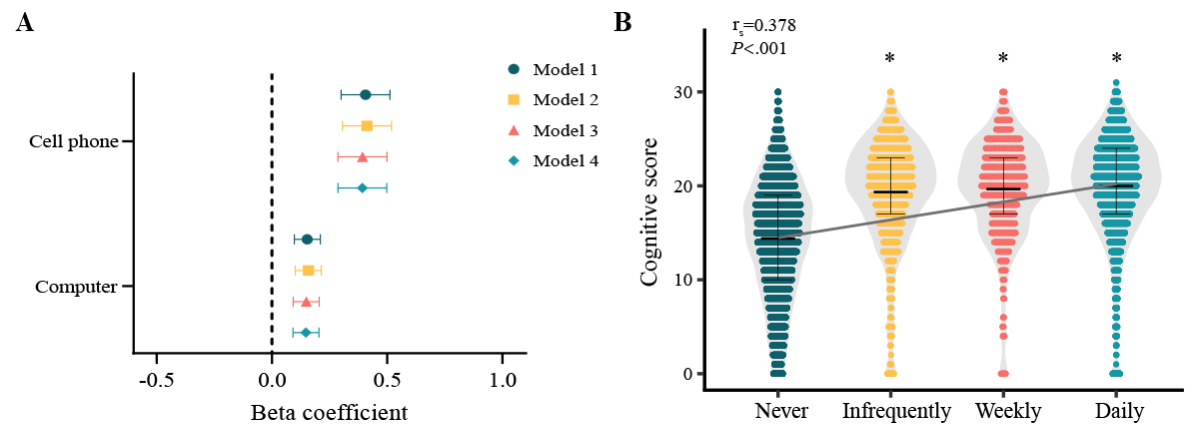
The influence on cognitive function of (A) internet device types (presented as beta coefficients and 95% CIs) and (B) frequency of internet use (assessed using Spearmans rank correlation). *P<.001, compared with individuals who never used the internet.

The fixed effects model (Figure 4A) suggests that, among middle-aged and older adults, internet users who use cell phones experience cognitive benefits (model 1: β=0.406, 95% CI 0.300 to 0.512, *P*<.001; model 2: β=0.412, 95% CI 0.306 to 0.519, *P*<.001; model 3: β=0.392, 95% CI 0.287 to 0.499, *P*<.001; model 4: β=0.398, 95% CI 0.283 to 0.495, *P*<.001). Furthermore, internet use on computer devices also has a positive influence on cognitive function (model 1: β=0.153, 95% CI 0.097 to 0.210, *P*<.001; model 2: β=0.158, 95% CI 0.101 to 0.214, *P*<.001; model 3: β=0.149, 95% CI 0.092 to 0.205, *P*<.001; model 4: β=0.147, 95% CI 0.091 to 0.204, *P*<.001), though to a lesser extent than cell phone–based internet use. This indicates that using cell phones as internet devices results in greater cognitive benefits than using computers. The proportion of mobile phone users among internet users increased dramatically over the past 5 years, from 64.5% (648/1005) to 99.3% (5463/5503), as detailed in Multimedia Appendix 4. However, the proportion of daily internet users only slightly increased, from 74.3% (747/1005) to 79.9% (4399/5503), as shown in Multimedia Appendix 4. Additionally, we used a Spearman rank correlation test to explore the association between internet use frequency and cognitive function. The results demonstrated a meaningful positive correlation between the frequency of internet use and cognitive function (r_s_=0.378, *P*<.001; Figure 4B). Moreover, individuals who reported using the internet infrequently, weekly, or daily scored higher on cognitive tests than those who never used it (*P*<.001). This suggests that, among middle-aged and older adults, the more frequent their internet use, the more beneficial it is for their cognitive function.

### Stratified and Interaction Analyses

**Figure 5:**
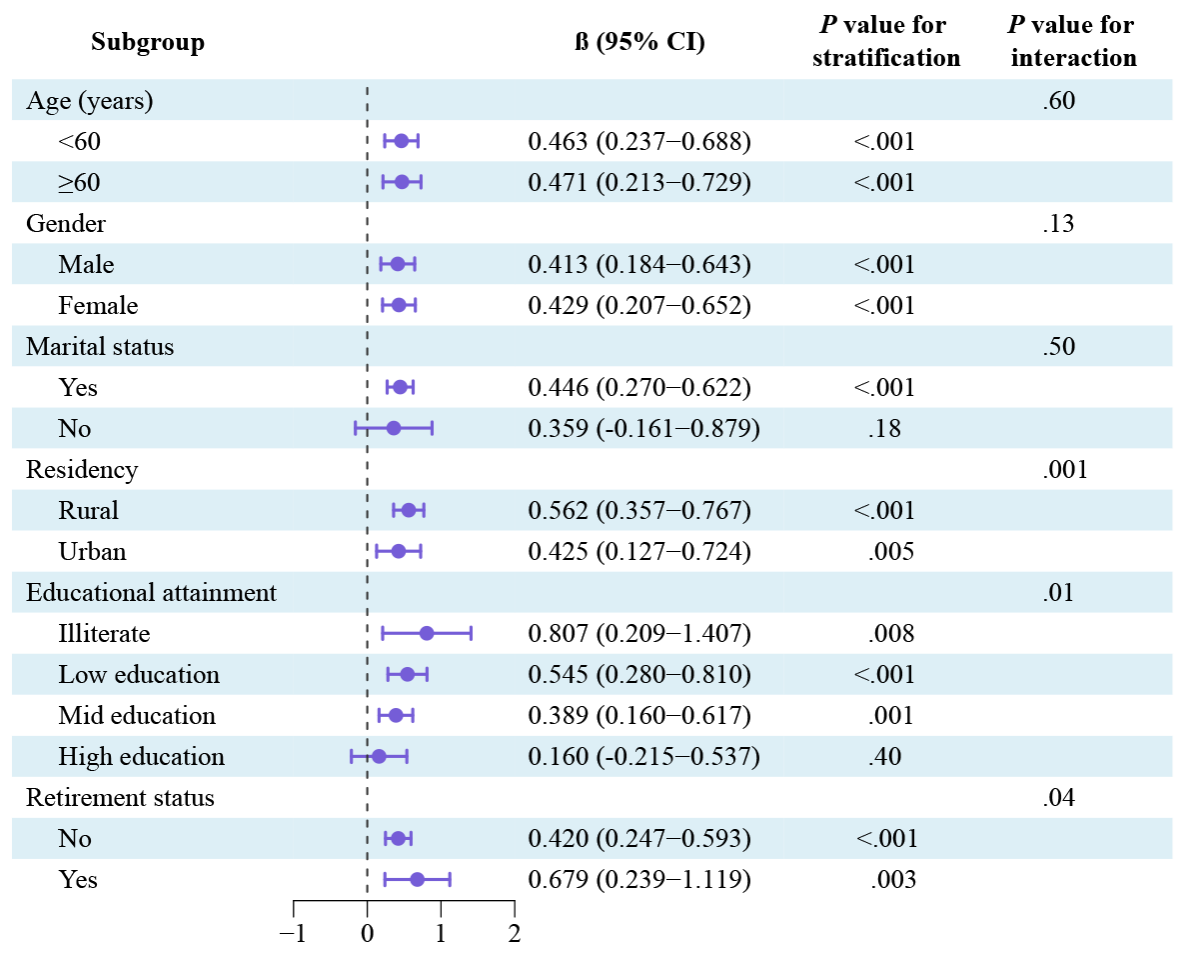
Association between internet use and cognitive function stratified by participant characteristics and adjusted for all other factors (prior cognitive score, age, gender, marital status, educational attainment, residency, retirement status, smoking, drinking, hypertension, diabetes, household income per capita), and the *P* value for the interaction was evaluated by including the variables’ cross-product term (internet use × baseline characteristics) in the same model.

Stratified analysis revealed that the positive correlation between internet use and cognitive function was similar across subgroups differentiated by age, gender, residency, and retirement status (Figure 5). In addition, internet use significantly affected individuals who were married (*P*<.001), while among those who were unmarried, there was only a trend present without a significant difference (*P*=.18). Interestingly, among middle-aged and older adults, there seemed to be a positive association between lower levels of educational attainment and higher levels of cognitive benefits gained from internet use (illiterate: β=0.807, 95% CI 0.209 to 1.407, *P*=.008; low education level: β=0.545, 95% CI 0.280 to 0.810, *P*<.001; mid education level: β=0.389, 95% CI 0.160 to 0.617, *P*=.001; high education level: β=0.160, 95% CI –0.215 to 0.537, *P*=.40). As the majority of middle-aged and older adults in China currently have low levels of education [27], there may be tremendous potential for cognitive benefits from internet use.

### Mediation Analyses

**Figure 6:**
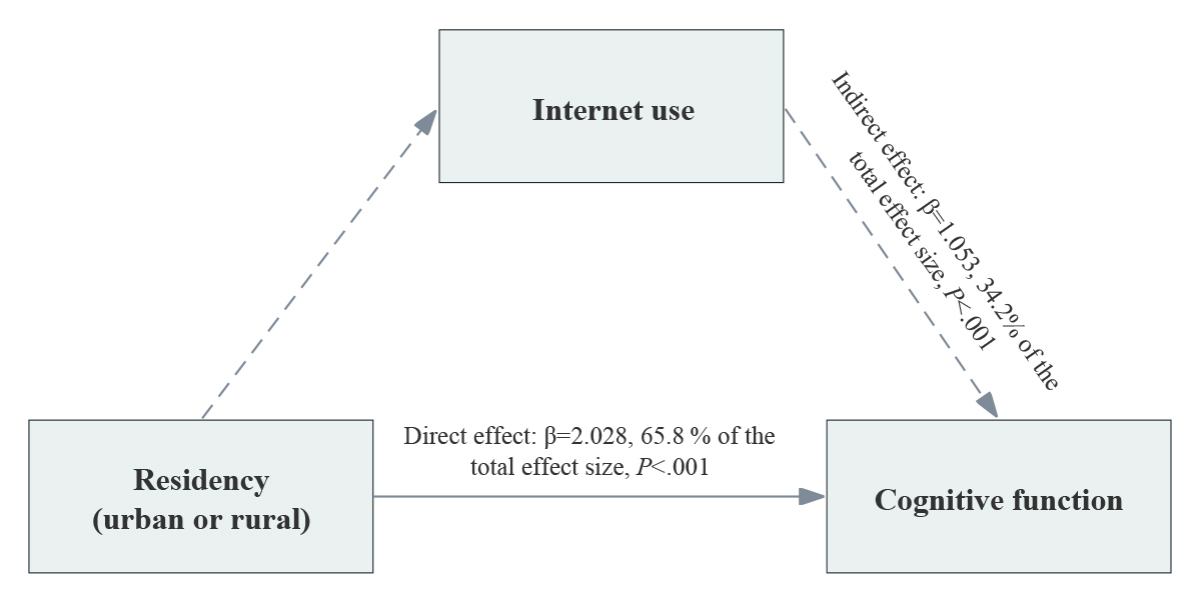
The mediation effect of internet use on the relationship between residential location and cognitive function using the Karlson-Holm-Breen (KHB) method.

Different areas of residence (urban or rural) among middle-aged and older adults resulted in notable differences in cognitive function (*P*<.001; Multimedia Appendix 5). In order to further explore the mediating effect of internet use on cognitive function in different residential areas, we used the KHB method for mediation analysis. As a result, internet use mediated 34.2% of the impact of residency on cognitive function (β=1.053, *P*<.001; Figure 6). This suggests that the differences in cognitive function between urban and rural areas can be partially attributed to variations in internet use.

### Sensitivity Analyses

**Figure 7:**
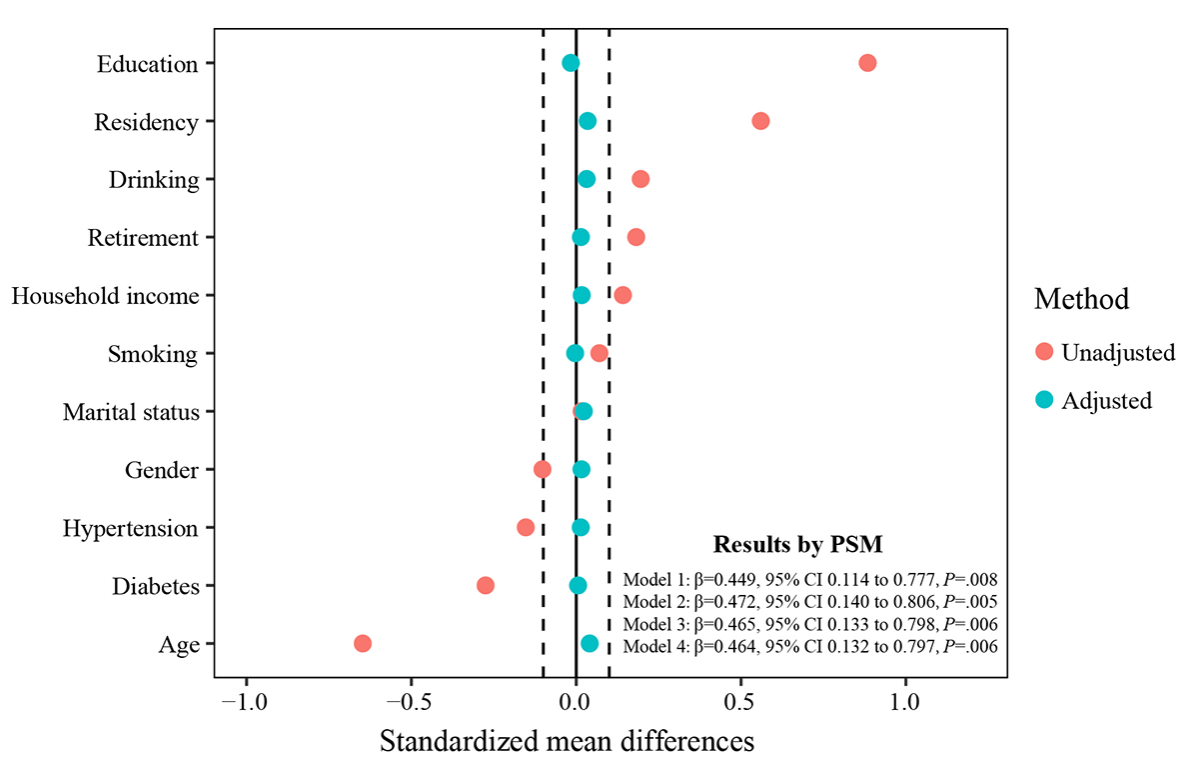
The effect of internet use on cognitive function after controlling covariate balance using propensity score matching (PSM), with each model adjusted after PSM.

Sensitivity analyses proved that the main findings of our study were robust. Following the implementation of PSM to achieve a balanced distribution of covariates (standardized mean difference<0.1) between internet users and nonusers, we obtained similar results in line with the fixed effects model (Figure 7). Moreover, we used IPTW to adjust for covariates, and the results were broadly consistent with the main analysis (Multimedia Appendix 6; model 1: β=0.559, 95% CI 0.142 to 0.976, *P*=.009; model 2: β=0.551, 95% CI 0.181 to 0.922, *P*=.004; model 3: β=0.514, 95% CI 0.167 to 0.861, *P*=.004; model 4: β=0.500, 95% CI 0.152 to 0.848, *P*=.005). In addition, we further used the OW to validate our main results (Multimedia Appendix 6; model 1: β=0.526, 95% CI 0.286 to 0.766, *P*<.001; model 2: β=0.551, 95% CI 0.312 to 0.790, *P*<.001; model 3: β=0.544, 95% CI 0.305 to 0.783, *P*<.001; model 4: β=0.542, 95% CI 0.303 to 0.781, *P*<.001). Last, when analyzing individuals who consistently used or did not use the internet for 5 years, the results of the GEE still demonstrated a positive relationship between internet use and cognitive function (Multimedia Appendix 7).

## Discussion

### Principal Findings

In this nationwide cohort study focusing on individuals aged 45 years and older, we found a positive relationship between internet use and cognitive function. Moreover, a higher frequency of internet use was associated with a lower risk of cognitive decline. Previous studies have suggested that using the internet can reduce social isolation, increase social interactions (both face to face and virtual or online), stimulate the acquisition of new knowledge, enhance cognitive demand for mastering novel skills using digital tools, and facilitate access to health and cultural information [28]. Individuals who use the internet may improve attention and psychomotor skills and enhance cognitive reserve through stimulation and reduction of depressive symptoms [29]. For middle-aged and older adults who use the internet, going online may help their brains use more efficient cognitive networks to counteract dementia [30]. Another explanation is that internet use is not only a form of cognitive stimulation but also a mode of social engagement. The sense of belonging created by online activities and the formation of social networks facilitate the brain's evolution and functional development [31]. These mechanisms partially account for the beneficial cognitive effects of internet use observed in our study.

Our study further explores the trends in the impact of aging on cognitive function among internet users and nonusers. In contrast to internet nonusers, we found that internet users showed a delay in age-related cognitive decline. Although we cannot ascertain a causal relationship from this observational study, we have reasons to speculate that internet use, as a cognitive-stimulating activity, may lead to beneficial physiological changes in the brain. With the progression of age, cognitive abilities such as attention, memory, executive function, language, and visuospatial skills prominently decline [32]. Additionally, the physiological changes in brain structure that accompany aging encompass alterations in neuronal structure, synaptic loss, and dysfunction of neuronal networks [33-35]. Research on brain plasticity provides strong support for the social modulation of cognition, as activity-related stimuli can induce angiogenesis, synaptogenesis, and neurogenesis in the brain [36,37]. Previous studies have not fully elucidated the cognitive benefits of internet use. Future studies could incorporate more detailed assessments of cognitive domains, neuroimaging data, or biomarkers to better understand the pathways through which internet use may influence cognitive function.

Our findings indicate that both computer-based and mobile-based internet use provide cognitive-enhancing effects, which is consistent with previous studies [38-40]. The research findings by Jin et al [38] revealed that middle-aged and older adults who own a cell phone or desktop computer at home experience a delay in cognitive decline. However, this study was based on the assessment of cell phone and desktop computer ownership, which is not as straightforward as our study in which we inquired about the specific devices used for internet access. Interestingly, our research findings suggest that middle-aged and older adults who use cell phones to access the internet have higher cognitive scores than those who use computers. This outcome might be explained by the fact that mobile phones have more advantages regarding operation and accessibility and are more user-friendly for older adults, so mobile phone users used the internet at a higher frequency and for longer durations. With the rapid development of social information technology, some mobile apps (such as WeChat and TikTok) have become increasingly vital to the daily lives of middle-aged and older adults, as they not only facilitate social communications and connections but also provide leisure and entertainment [41]. Meanwhile, these apps have lower operational barriers, making them more user-friendly. A previous study [42] showed that, as of 2012, older adults aged 65 years and older were more likely to own a mobile phone (69%) rather than a desktop computer (48%) or a laptop (32%). The study conducted by Liu et al [43] also suggested that the absence of using mobile social media platforms, such as WeChat and mobile payment systems, may be positively associated with cognitive impairments. Therefore, the impact of cell phone use on age-related cognitive decline among middle-aged and older adults deserves more attention.

The cognitive benefits of internet use among middle-aged and older adults with different educational levels seem to vary. The results of the stratified analysis suggest that internet use has a greater extent of cognitive benefits for those with lower educational attainment. Based on previous research, there continues to be controversy regarding the impact of education on age-related cognitive decline. Some research reports that education may slow the rate of age-related cognitive decline [44-46], while others [47,48] suggest that education has no significant effect on the rate of cognitive decline. Moreover, there have been studies indicating a relationship between higher levels of education and an accelerated cognitive decline, particularly in the domains of verbal memory, processing speed, and verbal fluency [49,50]. Although the impact of education on cognitive decline remains uncertain, internet use still holds enormous potential for improving cognition in individuals with low educational levels, particularly in China where 71.1% of older adults either have no formal education or have only received primary schooling [27]. On the other hand, although middle-aged and older adults with lower educational attainment receive great cognitive benefits from surfing the internet, they may face greater potential risks or unintended consequences (such as exposure to misinformation, online scams, and privacy concerns). Hence, ensuring internet safety for middle-aged and older adults, especially those with lower levels of education, constitutes a major challenge for developing a digital society in the future.

The Spearman rank correlation results in this study indicate that, among middle-aged and older adults, higher frequencies of internet use are associated with better cognitive performance. Although higher use frequency means better cognitive benefits, the impact of overuse of the internet cannot be ignored. Since specific durations of internet use for each individual could not be observed in this study, analyzing the cognitive impacts resulting from internet overuse was not feasible. More research is needed to understand the relationship between cognitive function and both the frequency and duration of internet use, especially in the case of overuse.

The phenomenon of urban-rural disparity in cognitive performance among middle-aged and older adults is prevalent in both lower-income and higher-income countries [51,52]. Previous research has indicated that cognitive functional disparities between urban and rural areas may be caused by various factors, including health care service resources, public education resources, early-life cognitive reserves, race, social class, social activities, and social engagement [53,54]. Our research revealed that internet use, as a tool for social participation, exerts a noteworthy mediating role in the cognitive function disparities between urban and rural areas. This indicates that promoting the use of the internet in rural regions could help to reduce the urban-rural gap in cognitive performance.

### Limitations

This study is not without limitations, and caution should be exercised when interpreting the findings. First, the study participants may not fully represent the middle-aged and older adult population in China, as the questionnaire survey was conducted only within the community and did not include public institutions such as nursing homes and hospitals. Therefore, the generalizability of our study findings primarily applies to healthy middle-aged and older adults, rather than frail older adults. Second, our analysis was based on a follow-up interval of approximately 5 years, and we cannot ascertain whether this correlation will endure over a more extended period. Third, the age of data and the changes in internet use over the past decade are factors that must be considered when interpreting the results of this study, as the pace of internet development and the expansion of internet access among older adults over the past 10 years may differ from what is expected in the next decade. Fourth, our approach to assessing cognitive function was not entirely based on specialized screening scales for cognitive impairment, such as the Mini-Mental State Examination or the Montreal Cognitive Assessment Scale. Therefore, caution must be exercised when interpreting the effects of internet use on mild cognitive impairment or Alzheimer disease. Fifth, due to the presence of loss to follow-up and unmeasured confounders, there may be bias in the association estimation. However, to ensure the robustness of the research data, we used various sensitivity analyses, including multiple linear regression analysis, GEE, PSM, IPTW, and OW, to validate the cognitive benefits of internet use. Sixth, our study did not directly establish a causal relationship between internet use and cognitive decline. However, the lower incidence of neurodegenerative diseases in persistent internet users indicated that internet use may prevent neurodegenerative diseases. Therefore, in order to prove the causal relationship between internet use and cognitive function, more research needs to be carried out, such as physiological mechanism research, instrumental variable analysis, and a Mendelian randomization study. Last, although the positive association between internet use and cognitive function was statistically significant after considering various potential confounders, there may still be some relevant variables, such as the apolipoprotein E4 genotype, that cannot be obtained from the survey questionnaire but could potentially influence the results.

### Conclusions

In conclusion, this study presents valuable evidence supporting a longitudinal protective association between internet use and cognitive function in middle-aged and older adults, with a higher frequency of internet use having a greater extent of cognitive benefit. As digital devices for internet access, cell phones may offer a potentially superior level of cognitive protection than computers. The cognitive gap among middle-aged and older adults in urban and rural areas can be partially attributed to internet use. This study suggests that internet use may be a promising cognitive stimulation activity that mitigates age-related cognitive decline and prevents neurodegenerative diseases. Although further research is needed to confirm the causal relationship, the findings highlight the potential benefits of promoting internet use among middle-aged and older adults, especially in rural areas, as a strategy to support cognitive health and potentially reduce the risk of dementia.

## Appendix

Multimedia Appendix 1 The questionnaire section on internet use and cognitive function in China Health and Retirement Longitudinal Study (CHARLS).

Multimedia Appendix 2 Cognitive scores of internet users and non-users at different waves.

Multimedia Appendix 3 The prevalence and incidence of neurodegenerative diseases (Alzheimer disease or Parkinson disease) in internet users and non-users during the 5-year period.

Multimedia Appendix 4 Changes in the proportion of internet users with different internet access devices and frequencies.

Multimedia Appendix 5 Cognitive scores of rural-urban participants at different waves.

Multimedia Appendix 6 The relationship between internet use and cognitive function in middle-aged and older adults was analyzed using inverse probability of treatment weighting (IPTW) and overlap weighting (OW) methods.

Multimedia Appendix 7 Differences in cognitive function between persistent internet users (n=671) and persistent non-users (n=7099) using generalized estimating equations (GEE) and multiple linear regression models.

## Abbreviations

CHARLS: China Health and Retirement Longitudinal Study
GEE: generalized estimating equations
IPTW: inverse probability of treatment weighting
KHB: Karlson-Holm-Breen method
OW: overlap weighting
PSM: propensity score matching

## Data Availability

The data sets used and analyzed during the study are available on the China Health and Retirement Longitudinal Study (CHARLS) website [55].
