# Supplementary material for: Relationship Between Internet Use and Cognitive Function Among Middle-Aged and Older Chinese Adults: 5-Year Longitudinal Study
Source: J Med Internet Res. 2024 Dec 2;26:e57301. doi: 10.2196/57301 (PMC11660964; doi:10.2196/57301)
Supplement: Multimedia Appendix 1 [file jmir_v26i1e57301_app1.docx]

**CHARLS Questionnaire-Internet Use Section**

1. Have you used the internet in the past month? Including chatting with mobile phone network, watching news, watching video, playing games, financial management and so on.

□ Yes

□ NO

1. How often in the last month did you do used the internet? Almost daily, almost every week, or not regularly?

□ Almost daily

□ Almost every week

□ Not regularly

1. Which types of devices do you use to access the internet?

□ Desktop computer

□ Laptop computer

□ Tablet computer

□ Cell phone

□ Other devices

**CHARLS Questionnaire-Cognitive Function Section**

[INTRO: First I would like to ask you some questions to check your memory and concentration.

Some of them may be easy and some may be hard.]

1. What is the year?

□ Correct

□ Error

□ Not assessed

1. What is the season of the year?

□ Correct

□ Error

□ Not assessed

1. What is the date?

□ Correct

□ Error

□ Not assessed

1. What is the day of the week?

□ Correct

□ Error

□ Not assessed

1. What is the month?

□ Correct

□ Error

□ Not assessed

[INTRO: please calculate 100 minus 7, and keep minus 7 continuously, tell me each answer you get from minus 7, until I say stop.]

1. 100 minus 7, and keep minus 7 continuously for five times

□ Record respondents' first responses

□ Record respondents' second responses

□ Record respondents' third responses

□ Record respondents' fourth responses

□ Record respondents' fifth responses

[INTRO: I am going to show you ten printed words. Read each word out loud as I show it to you. Later I will ask you to recall all ten words. ]

[PROGRAMMER: Show wordlist at a slow, steady rate, approximately one word every two seconds. Word sequence: Butter, Arm, Shore, Letter, Queen, Cabin, Pole, Ticket, Grass, Engine.]

1. Now please tell me the words you can recall.

□ Butter

□ Arm

□ Shore

□ Letter

□ Queen

□ Cabin

□ Pole

□ Shore

□ Ticket

□ Engine

□ Didn't recall any words

□ Refuse to recall

[PROGRAMMER: Score correct if A) There are two five-sided figures which intersect to form a foursided figure and B) All angles in the five sided figure much be preserved.]

1. Here is a drawing. Please copy the drawing on this paper.

□ Correct

□ Error

□ Not assessed

[INTRO: A few minutes ago I asked you to learn a list of ten words which you read one at a time from cards. Now I want you to try to recall as many of those 10 words as you can. OK, now tell me as many of those ten words as you can remember. ]

1. Please recall the words I showed you a few minutes ago.

□ Butter

□ Arm

□ Shore

□ Letter

□ Queen

□ Cabin

□ Pole

□ Shore

□ Ticket

□ Engine

□ Didn't recall any words

□ Refuse to recall

Note: The questionnaire has been translated in order to be easily accessible to readers in this publication. For more information about the original questionnaire, please refer to http://charls.pku.edu.cn/.
