# Supplementary material for: Relationship Between Internet Use and Cognitive Function Among Middle-Aged and Older Chinese Adults: 5-Year Longitudinal Study
Source: J Med Internet Res. 2024 Dec 2;26:e57301. doi: 10.2196/57301 (PMC11660964; doi:10.2196/57301)
Supplement: Multimedia Appendix 2 [file jmir_v26i1e57301_app2.docx]

**Table S1** Cognitive scores of internet users and non-users at different waves

|  | **Wave 3 (2015)** | | | **Wave 4 (2018)** | | | **Wave 5 (2020)** | | |
| --- | --- | --- | --- | --- | --- | --- | --- | --- | --- |
|  | **Non-users**  **(n=11,765)** | **Internet users**  **(n=1,005)** | ***P*** | **Non-users**  **(n=10,907)** | **Internet users**  **(n=1,863)** | ***P*** | **Non-users**  **(n=7,267)** | **Internet users**  **(n=5,503)** | ***P*** |
| **Mental intactness score** | 7.614±2.739 | 9.766±1.665 | <.001 | 6.463±2.981 | 8.729±2.238 | <.001 | 6.819±3.230 | 8.697±2.604 | <.001 |
| **Episodic memory score** | 7.028±3.469 | 10.075±2.957 | <.001 | 7.184±4.310 | 10.755±3.390 | <.001 | 9.074±4.951 | 12.302±4.272 | <.001 |
| **Total cognitive score** | 14.642±5.160 | 19.841±3.57 | <.001 | 13.647±6.383 | 19.484±4.596 | <.001 | 15.893±7.217 | 20.999±5.998 | <.001 |
