# Supplementary material for: Relationship Between Internet Use and Cognitive Function Among Middle-Aged and Older Chinese Adults: 5-Year Longitudinal Study
Source: J Med Internet Res. 2024 Dec 2;26:e57301. doi: 10.2196/57301 (PMC11660964; doi:10.2196/57301)
Supplement: Multimedia Appendix 3 [file jmir_v26i1e57301_app3.docx]

**Table S2** The prevalence of neurodegenerative diseases (Alzheimer disease or Parkinson disease) in internet users and non-users at follow-up time points.

|  | | **Wave 4 (2018)** | | | | **Wave 5 (2020)** | | | |
| --- | --- | --- | --- | --- | --- | --- | --- | --- | --- |
|  |  | **Non-users**  **(n=10,907)** | **Internet users**  **(n=1,863)** | **χ²** | ***P*** | **Non-users**  **(n=7,267)** | **Internet users**  **(n=5,503)** | **χ²** | ***P*** |
| **Neurodegenerative diseases** | **With** | 184  (1.7%) | 22  (1.2%) | 2.568 | 0.109 | 384  (5.3%) | 145  (2.6%) | 55.350 | ＜.001 |
|  | **Without** | 10,723  (98.3%) | 1,841  (98.8%) |  |  | 6,883  (94.7%) | 5,358  (97.4%) |  |  |

**Table S3** The incidence of neurodegenerative diseases (Alzheimer disease or Parkinson disease) in persistent internet users and persistent non-users over a 5-year period.

|  | | **3rd year after baseline (2018)** | | | | **5th year after baseline (2020)** | | | |
| --- | --- | --- | --- | --- | --- | --- | --- | --- | --- |
|  |  | **Non-users**  **(n=7,099)** | **Internet users**  **(n=671)** | **χ²** | ***P*** | **Non-users**  **(n=7,099)** | **Internet users**  **(n=671)** | **χ²** | ***P*** |
| **Neurodegenerative diseases** | **With** | 145  (2.0%) | 10  (1.5%) | 0.956 | 0.328 | 379  (5.3%) | 15  (2.2%) | 12.265 | ＜.001 |
|  | **Without** | 6,954  (98.0%) | 661  (98.5%) |  |  | 6,720  (94.7%) | 656  (97.8%) |  |  |
