# Supplementary material for: Relationship Between Internet Use and Cognitive Function Among Middle-Aged and Older Chinese Adults: 5-Year Longitudinal Study
Source: J Med Internet Res. 2024 Dec 2;26:e57301. doi: 10.2196/57301 (PMC11660964; doi:10.2196/57301)

**Figure S1** Changes in the proportion of internet users with different internet access devices and frequencies. (A) Changes in the proportion of internet users using cell phones and computers during the five-year follow-up period. (B) The percentage of internet users with different internet frequency changed during the five-year follow-up period.


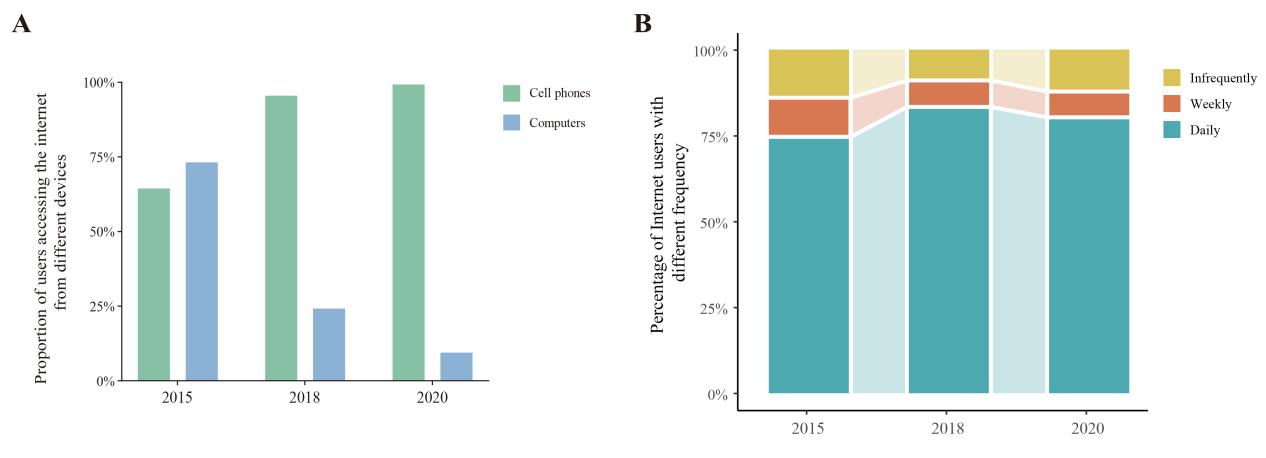

Supplement: Multimedia Appendix 4 [file jmir_v26i1e57301_app4.docx]
