# Supplementary material for: Relationship Between Internet Use and Cognitive Function Among Middle-Aged and Older Chinese Adults: 5-Year Longitudinal Study
Source: J Med Internet Res. 2024 Dec 2;26:e57301. doi: 10.2196/57301 (PMC11660964; doi:10.2196/57301)
Supplement: Multimedia Appendix 5 [file jmir_v26i1e57301_app5.docx]

**Table S4** Cognitive scores of rural-urban participants at different waves.

|  | **Wave 3 (2015)** | | |  | **Wave 4 (2018)** | | |  | **Wave 5 (2020)** | | |
| --- | --- | --- | --- | --- | --- | --- | --- | --- | --- | --- | --- |
|  | **Rural**  **(n=10,296)** | **Urban**  **(n=2,474)** | ***P*** |  | **Rural**  **(n=9,373)** | **Urban**  **(n=3,397)** | ***P*** |  | **Rural**  **(n=8,340)** | **Urban**  **(n=4,340)** | ***P*** |
| **Mental intactness score** | 7.512±2.774 | 8.910±2.226 | <.001 |  | 6.359±3.008 | 7.994±2.603 | <.001 |  | 7.260±3.147 | 8.344±2.933 | <.001 |
| **Episodic memory score** | 6.944±3.487 | 8.612±3.380 | <.001 |  | 7.137±4.338 | 9.274±4.077 | <.001 |  | 10.000±4.944 | 11.369±4.795 | <.001 |
| **Total cognitive score** | 14.456±5.209 | 17.522±4.633 | <.001 |  | 13.496±6.453 | 17.268±5.749 | <.001 |  | 17.260±7.153 | 19.713±6.949 | <.001 |
