# Supplementary material for: Relationship Between Internet Use and Cognitive Function Among Middle-Aged and Older Chinese Adults: 5-Year Longitudinal Study
Source: J Med Internet Res. 2024 Dec 2;26:e57301. doi: 10.2196/57301 (PMC11660964; doi:10.2196/57301)
Supplement: Multimedia Appendix 6 [file jmir_v26i1e57301_app6.docx]

**Table S5.** The relationship between internet use and cognitive function in middle-aged and older adults was analyzed using inverse probability of treatment weighting (IPTW) and overlap weighting (OW) methods.

|  | **IPTW** | |  | **OW** | |
| --- | --- | --- | --- | --- | --- |
|  | **β (95% CI)** | ***P*** |  | **β (95% CI)** | ***P*** |
| **Model 1** | 0.559 (0.142-0.976) | .009 |  | 0.526 (0.286-0.766) | <.001 |
| **Model 2** | 0.551 (0.181-0.922) | .004 |  | 0.551 (0.312-0.790) | <.001 |
| **Model 3** | 0.514 (0.167-0.861) | .004 |  | 0.544 (0.305-0.783) | <.001 |
| **Model 4** | 0.500 (0.152-0.848) | .005 |  | 0.542 (0.303-0.781) | <.001 |

Model 1 was adjusted for prior cognitive score; Model 2 was additionally adjusted for age, gender, marital status, educational attainment, residency, and retirement status; Model 3 was additionally adjusted for smoking, drinking, hypertension, and diabetes; Model 4 was additionally adjusted for household income per capita.
