# Supplementary material for: Relationship Between Internet Use and Cognitive Function Among Middle-Aged and Older Chinese Adults: 5-Year Longitudinal Study
Source: J Med Internet Res. 2024 Dec 2;26:e57301. doi: 10.2196/57301 (PMC11660964; doi:10.2196/57301)
Supplement: Multimedia Appendix 7 [file jmir_v26i1e57301_app7.docx]

**Table S6** Differences in cognitive function between persistent internet users (n=671) and persistent non-users (n=7,099) using generalized estimating equations (GEE) and multiple linear regression models. Model 1 was adjusted for baseline or prior cognitive test score; Model 2 was additionally adjusted for age, gender, marital status, educational attainment, residency, and retirement status; Model 3 was additionally adjusted for smoking, drinking, hypertension, and diabetes; Model 4 was additionally adjusted for household income per capita.

|  | **2015** | |  | **2018** | |  | **2020** | |  | **Total** | |
| --- | --- | --- | --- | --- | --- | --- | --- | --- | --- | --- | --- |
|  | **β (95% CI)** | ***P*** |  | **β (95% CI)** | ***P*** |  | **β (95% CI)** | ***P*** |  | **β (95% CI)** | ***P*** |
| **Model 1** | 7.049 (6.653- 7.445)^a^ | <.001 |  | 3.589 (3.181- 3.997) | <.001 |  | 2.346 (1.832-2.861) | <.001 |  | 1.979 (1.744-2.213) | <.001 |
| **Model 2** | 2.765 (2.353- 3.177) | <.001 |  | 1.099 (0.682- 1.517) | <.001 |  | 1.016 (0.496-1.535) | <.001 |  | 0.631 (0.380-0.882) | <.001 |
| **Model 3** | 2.738 (2.326-3.150) | <.001 |  | 1.120 (0.702-1.539) | <.001 |  | 0.908 (0.387-1.429) | <.001 |  | 0.574 (0.323-0.825) | <.001 |
| **Model 4** | 2.733 (2.318-3.148) | <.001 |  | 1.161 (0.740-1.582) | <.001 |  | 0.920 (0.397-1.444) | <.001 |  | 0.611 (0.358-0.864) | <.001 |

^a^represnts the crude model.
